# Ultrasound-guided preseptal hydrodissection with 5% dextrose in water for persistent botulinum toxin-associated ptosis: a technical protocol

**DOI:** 10.3389/fopht.2026.1792644

**Published:** 2026-05-18

**Authors:** King Hei Stanley Lam, Yonghyun Yoon, Jihyo Hwang, Hyemi Yu, Seungbeom Kim, Daniel Chiung-Jui Su

**Affiliations:** 1 Faculty of Medicine, The University of Hong Kong, Hong Kong, Hong Kong SAR, China; 2 Faculty of Medicine, The Chinese University of Hong Kong, Hong Kong, New Territories, Hong Kong SAR, China; 3 Department of Orthopaedic Surgery, Gangnam Sacred Heart Hospital, Hallym University College of Medicine, Seoul, Republic of Korea; 4 Incheon Terminal Orthopedic Surgery Clinic, Incheon, Republic of Korea; 5 The Board of Clinical Research, International Academy of Regenerative Medicine, Incheon, Republic of Korea; 6 Bio Plastic Surgery Clinic, Seoul, Republic of Korea; 7 Department of Pain Management, Miso Pain Clinic, Suwon, Gyeonggi, Republic of Korea; 8 Department of Pain Management and Rehabilitation, Chi Mei Medical Center, Tainan, Taiwan; 9 A Tempo Regeneration Centerfor Musicians, Tainan, Taiwan

**Keywords:** 5% dextrose, adverse effect, blepharoptosis, botulinum toxin, hydrodissection, marginal reflex distance, supraorbital nerve, ultrasound-guided

## Abstract

**Background:**

Botulinum toxin type A (BoNT-A) injections for glabellar rhytides can rarely lead to persistent blepharoptosis. Current management, including observation and α-adrenergic agonists, is often temporary, highlighting a need for definitive minimally invasive interventions.

**Methods:**

This article presents a novel, systematic protocol for ultrasound-guided preseptal hydrodissection. The technique utilizes high-frequency ultrasound with color Doppler to access the sub-orbicularis plane superficial to the orbital septum, where 20 mL of 5% dextrose in water (D5W) is delivered as low-pressure irrigational hydrodissection to establish a confined fluid layer.

**Results:**

Application of this protocol in two patients with persistent BoNT-A ptosis refractory to conventional care resulted in progressive improvement. The Marginal Reflex Distance 1 (MRD1) increased from 0 to 4 mm in one patient and normalized in the other, with gains maintained at 3–6 months without additional treatment and no adverse events.

**Conclusion:**

This work standardizes a technical protocol for a potential adjunctive intervention in persistent BoNT-A-associated ptosis. The findings are descriptive and hypothesis-generating, positioning the technique for consideration only after standard measures fail. Prospective controlled trials are required to establish efficacy and safety.

## 1 Introduction

Botulinum toxin type A (BoNT-A) is a cornerstone of minimally invasive aesthetic medicine for the treatment of dynamic facial rhytides (1). While its safety profile is well-established, the diffusion of the neurotoxin can lead to unintended muscular effects, most notably blepharoptosis, which is reported to occur in approximately 2%–5% of glabellar treatments (1–3). This complication results in functional visual field obstruction and significant psychosocial distress for patients, creating a persistent clinical challenge (4). The contemporary management paradigm for BoNT-A-associated blepharoptosis is well-defined, relying initially on observation as the pharmacological blockade wanes, supplemented by supportive measures such as eyelid taping or ptosis crutches (5–8). Pharmacologic intervention with topical α-adrenergic agonists (e.g., apraclonidine or oxymetazoline) can provide transient elevation of the eyelid through stimulation of Müller’s muscle, while surgical correction is reserved for exceptional, persistent, and functionally significant cases (5, 6, 8). Despite this structured approach, a distinct therapeutic gap remains for a subset of patients who experience prolonged, symptomatic ptosis. Existing options are often temporary, purely supportive, or inherently invasive, underscoring a clear unmet need for an effective, minimally invasive procedural intervention (7).

For the purposes of this protocol, persistent ptosis after BoNT−A is defined as functionally significant blepharoptosis—Marginal Reflex Distance 1 (MRD1) ≤ 2.0 mm or patient−reported visual field obstruction—present at >14 days following injection, despite an adequate trial of topical α−adrenergic agonist therapy (apraclonidine 0.5%–1% or oxymetazoline 0.1% for ≥48 h) with documented insufficient response (<1 mm MRD1 improvement and persistent functional complaint), and in the absence of spontaneous improvement or other non−BoNT−A causes on clinical evaluation.

Hydrodissection, the targeted injection of fluid to separate anatomical structures, is a foundational technique in interventional musculoskeletal and peripheral nerve medicine for releasing entrapped nerves (9). The application of 5% dextrose in water (D5W) as the injectate has garnered particular interest, with evidence suggesting it possesses not only a mechanical separating function but also neuromodulatory potential in the management of peripheral neuropathies (10–12). Building upon these principles, we hypothesize that targeted hydrodissection of the supraorbital and supratrochlear nerves—terminal branches of the ophthalmic division of the trigeminal nerve—may facilitate the recovery of eyelid function by mechanically dispersing the local fluid environment and modulating the perineural signaling milieu. Crucially, this protocol does not aim to biochemically reverse the presynaptic action of BoNT-A on motor nerve terminals. Instead, the proposed mechanism is mechanical and modulatory: the establishment of a fluid plane within the sub-orbicularis preseptal space may (i) disperse local tissue edema and inflammatory mediators that could theoretically impair residual neuromuscular function, (ii) reduce mechanical impedance to residual levator function by separating fascial planes, and (iii) modulate perineural signaling of the supraorbital and supratrochlear nerves, which are sensory branches of the trigeminal nerve. The potential influence on sensory afferents is hypothesized to indirectly affect motor recovery through reflex modulation or reduced sympathetically mediated effects on the local tissue environment, though this remains speculative. This remains a mechanistic hypothesis to be tested.

This Methods article presents a novel, systematic protocol for ultrasound-guided preseptal irrigational hydrodissection for persistent BoNT-A-induced blepharoptosis. The primary objective is to provide a detailed, reproducible, and safety-focused procedural guide. We detail a step-by-step technique utilizing an extra-orbital, preseptal (sub-orbicularis) approach to establish a fluid plane strictly superficial to the orbital septum. This protocol differs from existing perineural injection techniques in several critical aspects: (1) it employs a low-pressure irrigational hydrodissection approach (20 mL total volume) rather than focal perineural injection (typically 1–5 mL); (2) the objective is establishment of a continuous fluid plane for tissue mobilization rather than isolated nerve decompression; and (3) the technique is strictly confined to the preseptal space superficial to the orbital septum, an anatomical plane not previously described as a target for hydrodissection in this clinical context.

Ultrasound guidance is employed principally for real-time plane control and safety assurance, ensuring continuous in-plane needle visualization, utilizing color Doppler to avoid vasculature, and strictly confining fluid anterior to the orbital septum. The protocol is subsequently illustrated through its application in two representative cases, which serve to demonstrate feasibility and preliminary outcomes. All discussions regarding potential mechanisms and efficacy are presented as hypothesis-generating, consistent with the aim of introducing a standardized technique to address a defined clinical need.

## 2 Materials and equipment

The following materials and equipment were used to perform the ultrasound-guided preseptal hydrodissection procedure (**Table 1**).

**Table 1:** Materials and equipment used for ultrasound-guided preseptal hydrodissection.

Category	Item	Specifications/Details
Ultrasound system	Ultrasound machine	High-resolution system with real-time imaging and color Doppler functionality.
	Linear transducer	High-frequency linear array (8–15 MHz). *For this study:* Alpinion XC90 elite system with an 8-MHz probe.
	Key settings	Depth: ~2 cm; focal zone aligned to the supraorbital rim.
Procedure consumables	Needle	25-gauge, 1.5-inch hypodermic needle.
	Injectate	20 mL of 5% dextrose in water (D5W), prepared aseptically. *Note:* No local anesthetic added.
Skin preparation and other		Chlorhexidine or povidone-iodine solution, sterile gloves, gauze, adhesive dressing, normal saline, acoustic coupling gel

## 3 Methods

### 3.1 Objectives

The primary objective of this ultrasound-guided protocol is to perform a precise, extra-orbital hydrodissection to establish a continuous fluid plane within the sub-orbicularis preseptal space—that is, superficial to the orbital septum. The procedure aims to anteriorly disperse local tissue fluid and modulate the perineural environment of the supraorbital and supratrochlear nerves, with the goal of facilitating functional recovery in cases of persistent BoNT-A-induced blepharoptosis. A critical safety objective is the strict containment of injectate anterior to the orbital septum to avoid posterior (orbital) spread.

### 3.2 Step-by-step procedural protocol

#### 3.2.1 Patient preparation and positioning

1. Position the patient supine on the procedure table.
2. Obtain and document informed consent, explicitly discussing the investigational nature of the procedure, potential benefits, and risks.
3. Prepare the skin of the supraorbital region and upper eyelid thoroughly with an antiseptic solution (e.g., chlorhexidine or povidone-iodine).

#### 3.2.2 Sonographic landmark identification and plane targeting

**Figure 1:**
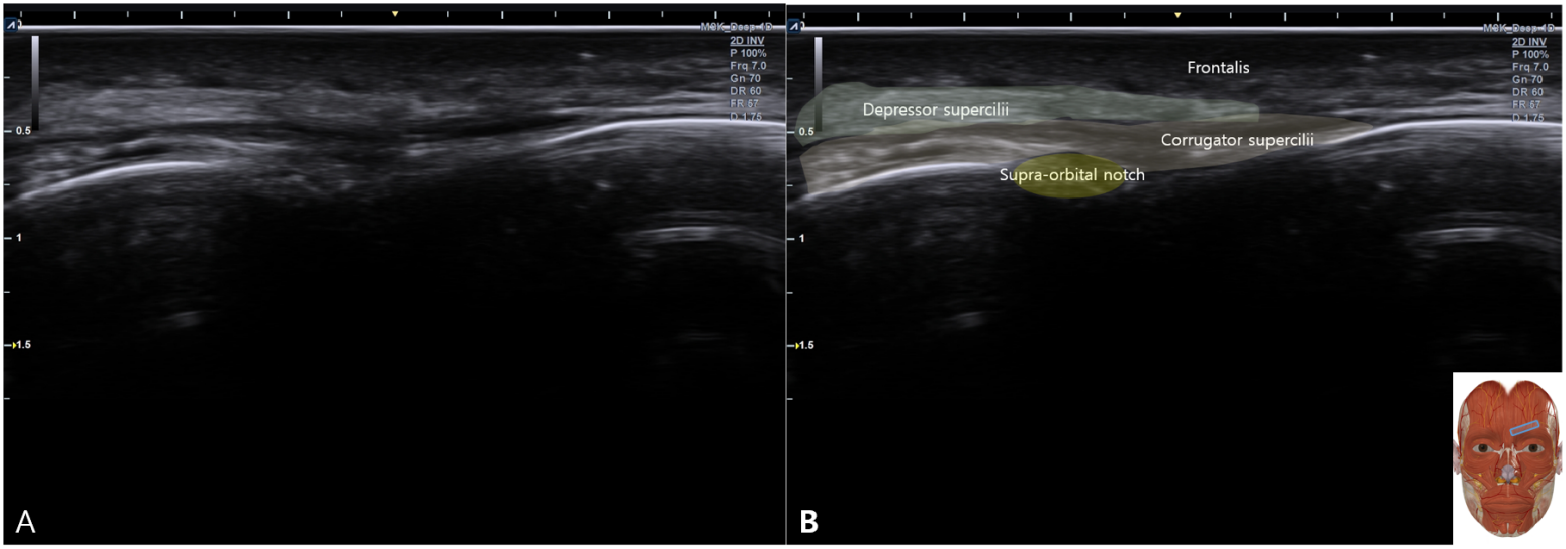
Ultrasound anatomy of the orbital region. **(A)** 2D image showing the supraorbital foramen. **(B)** Corresponding schematic diagram.

**Figure 2:**
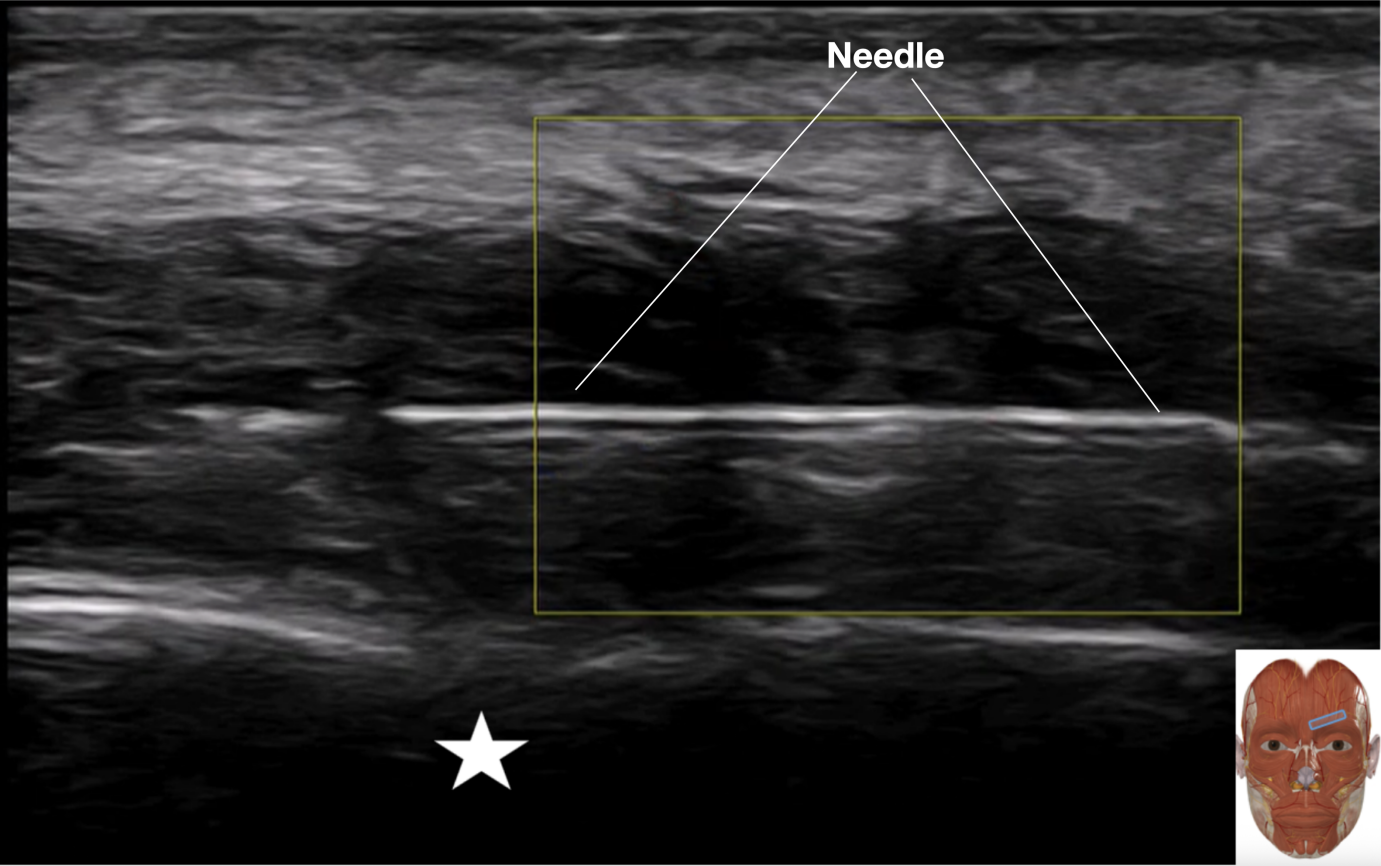
Ultrasound image showing needle position during hydrodissection within the preseptal (sub-orbicularis) plane, superficial to the septum (white star: supraorbital notch).

1. Apply acoustic coupling gel and place the high-frequency linear transducer (8–15 MHz) in an oblique orientation along the supraorbital rim.
2. Identify the primary bony landmark, the supraorbital notch/foramen, visualized as a discontinuity in the hyperechoic bony cortex with acoustic shadowing (**Figure 1A**).
3. Visualize the overlying muscular anatomy, including the corrugator supercilii, depressor supercilii, and frontalis muscles (**Figure 1B**).
4. Mandatory pre-needle step: Activate color Doppler to map the course of the supraorbital and supratrochlear arteries. Select an avascular pathway for needle advancement to minimize hematoma risk (**Figure 2**).

#### 3.2.3 Needle approach and hydrodissection execution

**Figure 3:**
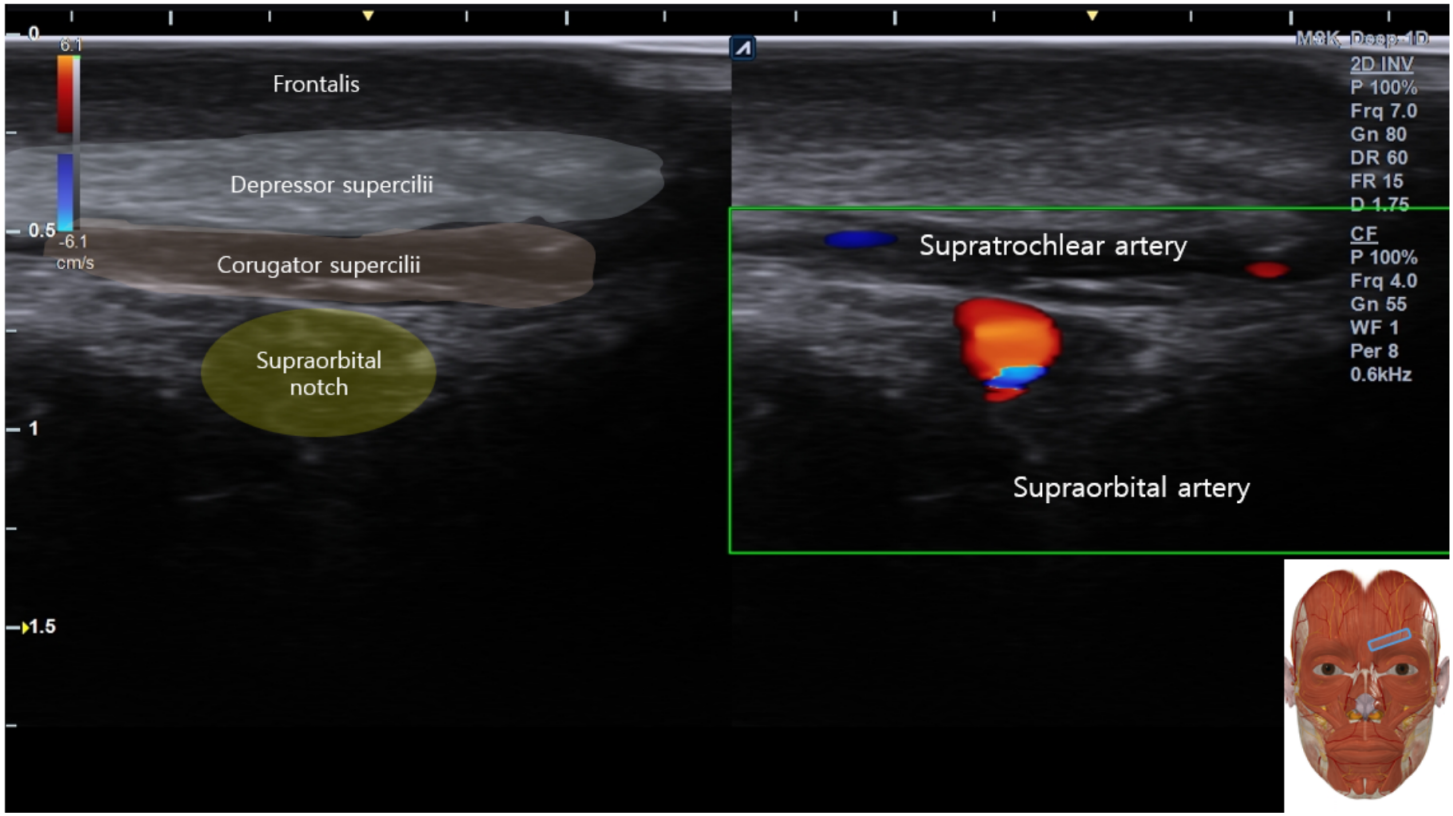
Schematic drawing of the supraorbital foramen anatomy in live dual and appearance of blood vessels confirmed in Doppler.

1. Using a strict in-plane ultrasound technique, advance a 25-gauge, 1.5-inch needle from the lateral aspect toward the medial supraorbital region.
2. Under continuous visualization, direct the needle tip into the sub-orbicularis plane, ensuring its position is anterior to the hyperechoic line of the orbital septum (**Figure 3**).
3. Plane verification: Perform a low-pressure test injection of 0.5–1 mL of D5W. Correct preseptal placement is confirmed by visualization of a thin anechoic layer expanding superficial to the septum.
4. Irrigational hydrodissection: Upon confirmation, proceed with slow, low-pressure infusion of D5W. The injectate should be observed in real time as an expanding anechoic pocket, hydrodissecting the fascial planes associated with the corrugator supercilii muscle and establishing a continuous preseptal fluid layer.
5. Termination point: Cease injection when a stable, continuous preseptal anechoic layer is established, the predefined fluid endpoint is achieved, and the patient remains comfortable.

#### 3.2.4 Post-procedure assessment

1. Monitor the patient for 10–15 min.
2. Assess for any immediate complications: test extraocular movements, inquire about diplopia or visual changes, and inspect for proptosis or excessive periorbital tension.
3. Obtain standardized frontal photographs in neutral gaze for documentation.
4. Provide the patient with written aftercare instructions, including warning signs that necessitate immediate medical attention (e.g., sudden vision change, severe pain, and marked swelling).

### 3.3 Validation of the method and procedural endpoints

The validity of the technique is confirmed intraoperatively by achieving two predefined, ultrasound-verified endpoints:

1. Primary endpoint (technical success): Establishment of a sustained, continuous anechoic (fluid) layer within the preseptal fascial plane.
2. Primary safety endpoint: Absence of fluid displacement posterior to the hyperechoic line of the orbital septum. Any posterior spread is an immediate stop criterion.

The procedure is considered technically complete and successful only when both endpoints are met simultaneously. The empirical volume of 20 mL of D5W was determined based on prior experience with superficial fascial hydrodissection, representing the volume consistently required to achieve the predefined sonographic endpoint—establishment of a continuous anechoic fluid layer spanning the target anatomical region (from the supraorbital notch medially to the lateral aspect of the eyebrow) (13–15). This volume is consistent with published hydrodissection protocols for superficial fascial planes where irrigational rather than focal injection is employed. Volumes less than 20 mL were observed in preliminary cases to result in incomplete or discontinuous fluid spread, failing to achieve the primary technical endpoint of a sustained continuous preseptal layer. Conversely, volumes exceeding 20 mL did not confer additional sonographic benefit but increased patient discomfort. Thus, 20 mL was selected as the empirically determined volume that reliably achieves the technical endpoint while maintaining patient comfort.

### 3.4 Troubleshooting, pitfalls, and safety management

**Anticipated challenges and solutions:**

- Difficulty visualizing the plane: Ensure optimal machine settings (depth ~2 cm, focal zone on bone). Use copious coupling gel and adjust transducer angle. If the plane remains unclear, abort the procedure.
- Vascular encounter: If Doppler mapping shows a vessel in the planned needle path, immediately retract and re-angle the needle to identify an avascular window. Avoid applying excessive compression with the transducer or during ultrasound guidance, as compression can temporarily occlude small vessels and mask their presence.
- Patient discomfort: A transient dull ache is common. A sharp pain surge indicates potential needle contact with periosteum or a nerve branch—stop injecting and slightly withdraw the needle.

Critical stop-rules (immediate procedure cessation):

- Surge in patient pain.
- Loss of laminar fluid spread or development of tense edema.
- Any sonographic sign of fluid tracking posterior to the orbital septum.
- Onset of visual symptoms reported by the patient.

**Contraindications:**

This procedure should not be performed in patients with active local infection; suspected orbital compartment syndrome; uncontrolled bleeding diathesis or therapeutic anticoagulation without appropriate risk mitigation; acute unexplained vision changes; or inability to cooperate with the procedure.

## 4 (Anticipated) Results

### 4.1 Expected technical and sonographic outcomes

When the protocol is correctly followed, the operator should achieve several clear, real-time ultrasound endpoints confirming successful preseptal hydrodissection. The primary technical goal is the establishment of a sustained, continuous anechoic fluid layer within the sub-orbicularis space, superficial to the hyperechoic orbital septum, as illustrated in **Figure 2**.

Crucially, this fluid spread will be confined anteriorly, with no sonographic evidence of posterior displacement into the orbital compartment. Throughout the procedure, key anatomical landmarks—including the supraorbital notch (**Figure 1**) and associated neurovascular structures pre-mapped with color Doppler (**Figure 3**)—must remain clearly identifiable to ensure ongoing safety and precision. The successful creation of this preseptal fluid layer is hypothesized to modulate the local tissue environment to facilitate functional recovery.

### 4.2 Example of application and effectiveness: clinical case illustrations

To demonstrate the application of this protocol and its potential effectiveness, we present the following two clinical cases of persistent BoNT-A-associated ptosis. These cases are presented as examples of feasibility and technical application, not as definitive evidence of efficacy. The authors acknowledge that a larger case series would be desirable to further characterize the technique’s safety profile and variability of outcomes; such work is currently ongoing.

#### 4.2.1 Clinical outcomes

Two patients with persistent, functionally significant blepharoptosis unresponsive to conventional management underwent three sessions of the described hydrodissection.

**Figure 4:**
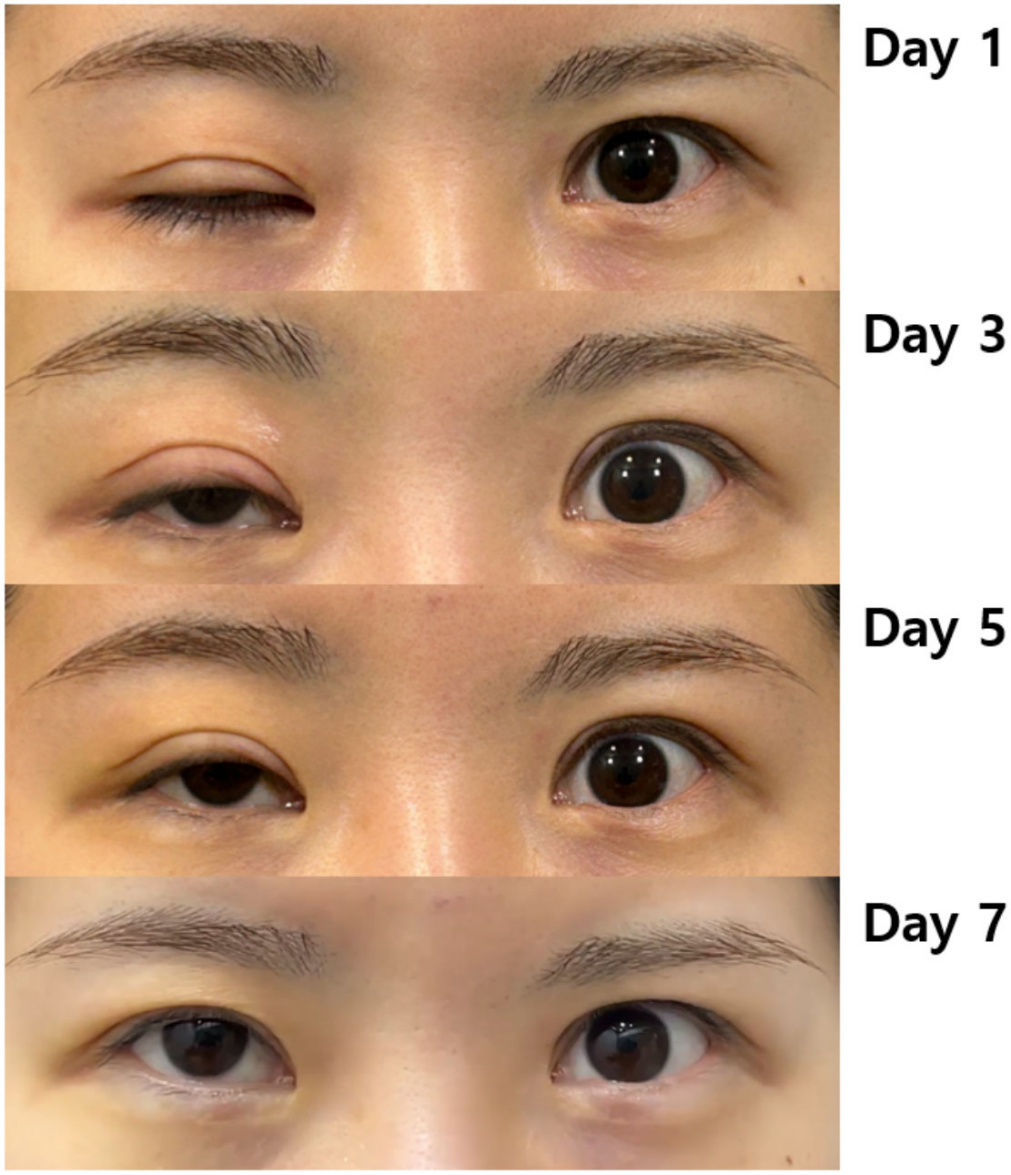
Changes in ptosis according to treatment progress in Case 1.

**Figure 5:**
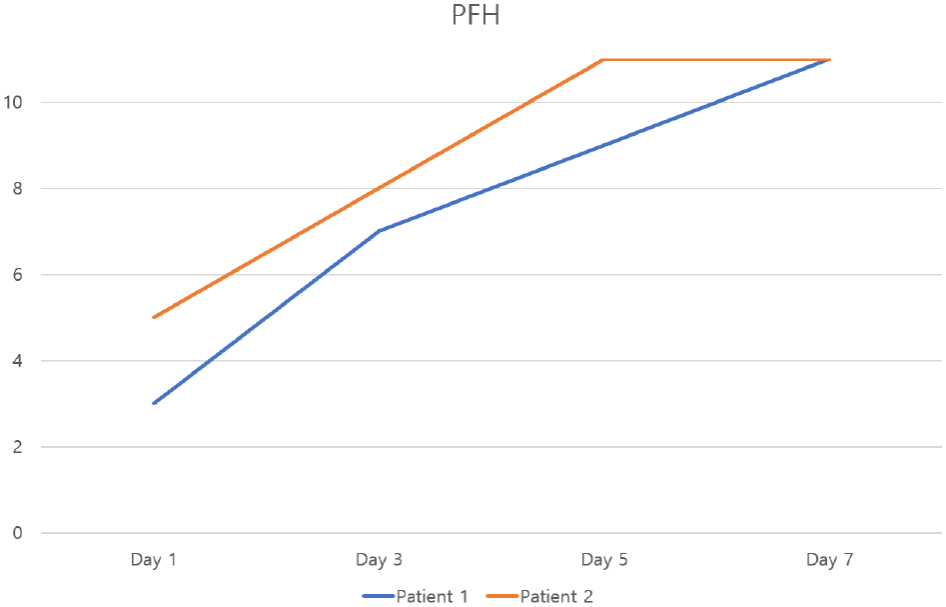
Changes in PFH over time in patients 1 and 2 (PFH: palpebral fissure height).

**Figure 6:**
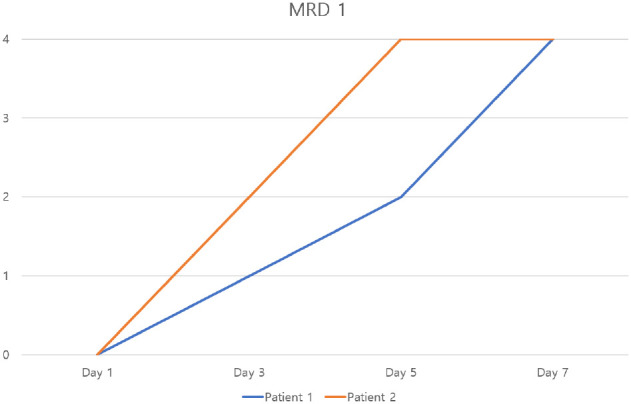
Changes in MRD1 over time in patients 1 and 2 (MRD1: Marginal Reflex Distance 1).

**Figure 7:**
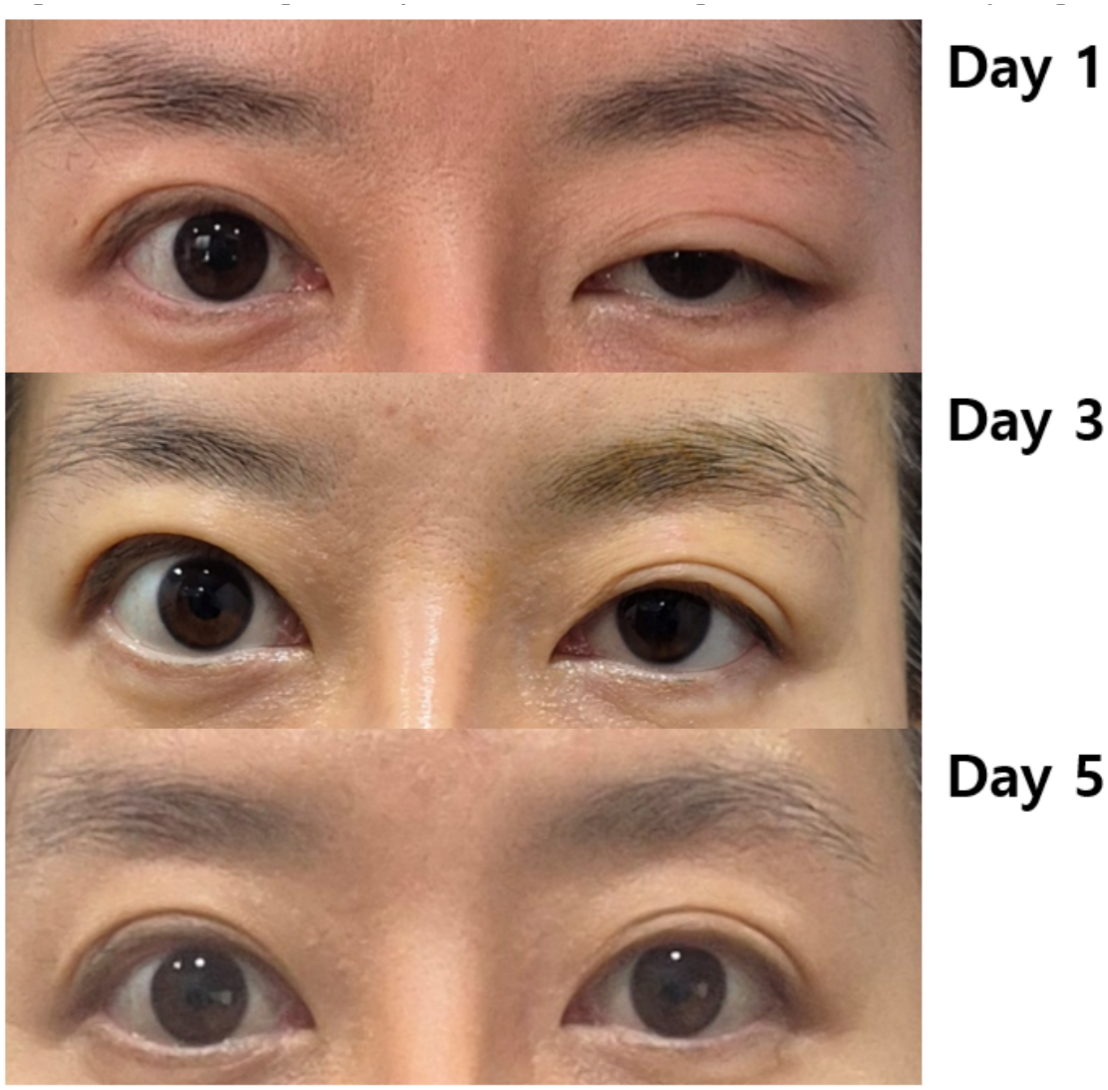
Changes in ptosis according to treatment progress in Case 2.

- Case 1: A woman in her 20s presented with a left-sided MRD1 of 0 mm. Following the procedure, her MRD1 improved to 4 mm, with normalization of palpebral fissure height (PFH). This improvement was maintained at the 6-month follow-up (**Figures 4**–**6**).
- Case 2: A woman in her 40s presented with a right-sided MRD1 of 0 mm. Following treatment, her MRD1 and PFH normalized, allowing discontinuation of an eye patch, with results stable at 6 months (**Figures 6**, **7**).
- Objective data for both patients are summarized in **Table 2**. No adverse events, such as hematoma, orbital injury, or infection, were observed in either case.

**Table 2:** Standardized case summary.

Category	Variable	Case 1 (Female, 20s)	Case 2 (Female, 40s)
Baseline	Comorbidities	None	None
	Symptom duration at presentation	10 days	1 month
	Prior interventions (all prior to HD)	Apraclonidine, periocular laser/radiofrequency, massage, Müller muscle surgery	Apraclonidine, periocular laser, observation, self-massage
Procedure	Target plane and guidance	Preseptal; continuous in-plane ultrasound and color Doppler	Preseptal; continuous in-plane ultrasound and color Doppler
	Needle and injectate	25-gauge; 5% dextrose in water (D5W)	25-gauge; 5% dextrose in water (D5W)
	Total volume and sessions	20 mL, every other day × 3 sessions	20 mL, every other day × 3 sessions
Objective outcomes	MRD1 (baseline → key follow-up)	0 mm → 4 mm (Week 1); maintained at Months 3 and 6	0 mm → within typical range (Week 1); maintained at Months 3 and 6
	PFH	Normalized and maintained	Normalized and maintained
Follow-up and safety	Scheduled follow-ups	Day 1, W1, W4, M3, M6 (in-person)	Day 1, W1, W4, M3, M6 (in-person)
	Additional procedures after HD	No	No
	Adverse events/Recurrence	None/None	None/None

#### 4.2.2 Interpretation of example outcomes

These cases serve as illustrative examples of the protocol’s successful application. The observed progressive improvement and durable results at 3–6 months post-procedure align with the hypothesized mechanism of action; however, given the small sample size (*n* = 2) and uncontrolled design, these findings cannot be distinguished from the natural history of spontaneous recovery. It should be noted that Case 1 was treated at 10 days post-BoNT-A injection, a time point that overlaps with the period during which spontaneous resolution of ptosis may begin to occur in some patients. This timing represents a limitation of the illustrative case and underscores the importance of establishing a stricter definition of persistence (≥14 days) as proposed in this protocol. These findings should be interpreted as preliminary and hypothesis-generating. The level of evidence is consistent with a case series (Level IV evidence) and does not support definitive conclusions regarding efficacy. The absence of complications in these examples underscores the importance of adhering to the ultrasound-guided safety measures detailed in the protocol.

### 4.3 Advantages, limitations, and potential pitfalls

- Primary advantages: The technique is minimally invasive, utilizes real-time ultrasound for precision and safety, and targets a specific anatomical plane to avoid orbital structures.
- Key limitations and pitfalls:

Sample size and generalizability: The most substantial limitation of this work is the very small sample size (*n* = 2). This precludes any statistical inference regarding efficacy and limits the generalizability of the findings to the broader population of patients with BoNT-A-associated ptosis. Outcomes in future patients may differ from those reported here.

Study design: This is an uncontrolled, observational description of a technical protocol with illustrative cases. The absence of a control group (e.g., sham procedure, observation-only, or alternative treatment) means that the observed improvements cannot be definitively attributed to the intervention rather than to the natural history of spontaneous neuromuscular recovery, which typically occurs over weeks to months following BoNT-A injection.

*Operator dependence:* The procedure is highly operator-dependent, requiring proficiency in high-frequency ultrasound imaging of the orbital region, accurate identification of the preseptal plane, and precise needle control. Reproducibility across different practitioners and clinical settings has not been established.

Volume rationale: The 20-mL volume was empirically derived based on prior experience with superficial fascial hydrodissection and sonographic endpoints rather than through formal dose-finding methodology. The optimal volume—balancing therapeutic effect against safety considerations—remains unknown.

Outcome measurement: Validated patient-reported outcome measures (e.g., quality-of-life instruments specific to periocular function) were not systematically collected. Objective measurements (MRD1 and PFH), while quantifiable, capture only one dimension of clinical improvement and may not fully reflect patient-perceived benefit.

Safety data: While no adverse events occurred in these two cases, the small sample size provides limited power to detect rare but potentially serious complications, such as orbital penetration, retrobulbar hemorrhage, or globe injury.

Potential for posterior spread: The most critical procedural risk—posterior fluid spread into the orbital compartment—remains a theoretical concern despite strict adherence to stop-rules. The safety margin of this technique has not been established in larger cohorts.

- Troubleshooting: As detailed in Section 3.4, immediate cessation of injection and needle repositioning is required upon encountering a pain surge, loss of fluid containment, or any visual symptoms.

## 5 Discussion

This report details a novel, systematic protocol for ultrasound-guided preseptal hydrodissection with 5% dextrose in water for the management of persistent BoNT-A-associated blepharoptosis. The primary contribution of this work is the meticulous description of a reproducible technique that prioritizes anatomical precision and safety through real-time sonographic control. The application of this protocol in two illustrative cases, resulting in improved and durable eyelid elevation, serves as a preliminary demonstration of its potential clinical utility and feasibility. It is crucial to emphasize that this technique is framed as a potential adjunctive intervention within a structured treatment hierarchy, to be considered only after conventional first-line measures have been exhausted.

### 5.1 Technical rationale and procedural advantages

The core technical innovation of this protocol lies in its targeted, extra-orbital approach. By confining the hydrodissection to the sub-orbicularis preseptal plane superficial to the orbital septum, the procedure aims to influence the perineural environment of the supraorbital and supratrochlear nerves while explicitly avoiding the orbital compartment. This approach differs substantively from conventional perineural injection techniques. Traditional hydrodissection for peripheral nerve entrapment typically involves focal injection of 1–5 mL of solution directly surrounding an isolated nerve trunk under ultrasound guidance (9). In contrast, our protocol employs low-pressure irrigational hydrodissection with a larger volume (20 mL) to establish a continuous fluid plane across the entire sub-orbicularis space, with the objective of mobilizing fascial planes and dispersing local tissue factors rather than solely decompressing a single nerve. Additionally, the preseptal plane—superficial to the orbital septum—represents a distinct anatomical target not previously described for hydrodissection in this clinical context. The mechanistic hypothesis underpinning this approach is distinct from that of BoNT-A. While BoNT-A exerts its effect through presynaptic blockade of acetylcholine release at motor nerve terminals, this hydrodissection technique does not propose to reverse that pharmacologic effect. Rather, the hypothesized benefit is mechanical (reduction of fascial impedance and redistribution of local fluid) and potentially neuromodulatory (influence on sensory afferents that may affect the local neuromuscular environment). These mechanisms are distinct and complementary to the pathophysiology of BoNT-A. The indispensable role of high-frequency ultrasound guidance cannot be overstated. It enables the precise identification of variable neurovascular anatomy, provides a real-time visual roadmap for needle advancement, and offers continuous feedback on fluid spread. The mandatory use of color Doppler to map vessels and the predefined sonographic endpoints (continuous preseptal layer, no posterior spread) constitute the fundamental safety pillars of this technique. These features collectively enhance the procedure’s reproducibility and mitigate risks in a highly sensitive anatomical region.

### 5.2 Clinical context and positioning

The management of BoNT-A-induced ptosis follows a well-established escalating pathway, beginning with observation, progressing to supportive measures and pharmacologic therapy with topical α-adrenergic agonists (e.g., apraclonidine), and reserving surgical correction for permanent deficits. The protocol described herein is not intended to disrupt this pathway but to offer a minimally invasive, potential adjunct within it. It may be considered for a select subset of patients who experience persistent, functionally significant symptoms despite first-line therapies and for whom the waiting period for spontaneous recovery or the prospect of surgery is unacceptable. This positioning is hypothesis-generating and requires validation through comparative studies.

### 5.3 Mechanistic considerations and limitations

The precise mechanism by which this hydrodissection technique may facilitate recovery remains speculative and is presented as a hypothesis to inform future research. We do not propose a reversal of BoNT-A’s presynaptic action. Instead, the effect is hypothesized to be multifactorial, potentially involving the mechanical establishment of a low-resistance glide layer, anterior redistribution of local tissue fluid, and modulation of the perineural milieu by the hypertonic dextrose solution. It is important to clarify that this technique targets sensory nerve structures (supraorbital and supratrochlear nerves) rather than the motor nerve terminals affected by BoNT-A. The hypothesized mechanism is therefore indirect: modulation of sensory afferents may influence local tissue physiology, including vascular tone, edema resolution, and potentially reflex modulation of motor function, though this remains speculative and requires basic science investigation. These proposed mechanisms are derived from the documented neuromodulatory and hydrodissective effects of perineural D5W in other peripheral nerve applications (9, 16).

This report has several important limitations that must be acknowledged to contextualize the findings:

Small sample size and descriptive nature: The most significant constraint is the very small sample size (*n* = 2). This work is fundamentally descriptive and hypothesis-generating, intended to introduce a standardized technical protocol rather than to establish therapeutic efficacy. The two cases presented serve as illustrative examples of technical feasibility, not as evidence of reproducible clinical outcomes.

Lack of control group and confounding by natural history: The uncontrolled, observational design represents a critical limitation. BoNT-A-associated ptosis has a well-documented natural history of spontaneous resolution as the neurotoxin effect wears off over weeks to months. Without a control group receiving observation alone, sham treatment, or an alternative intervention, the observed improvements in eyelid position cannot be statistically distinguished from the expected course of spontaneous neuromuscular recovery. This confounding factor precludes any definitive attribution of clinical improvement to the hydrodissection procedure itself. Additionally, the timing of intervention in Case 1 (10 days post-BoNT-A) represents a confounding factor, as this period may coincide with the early phase of spontaneous neuromuscular recovery in some patients.

Operator dependence and reproducibility: The technique requires specialized proficiency in high-frequency ultrasound imaging of the orbital region and precise needle control under real-time guidance. Reproducibility across different operators, clinical settings, and patient populations has not been established. Outcomes may vary substantially based on operator experience.

Empirically derived volume: The 20-mL volume was empirically determined based on prior experience with superficial fascial hydrodissection and the sonographic endpoint of achieving a continuous fluid layer across the target anatomical region. Formal dose-finding studies have not been conducted, and the minimal effective volume—or whether volume correlates with therapeutic response—remains unknown.

Incomplete outcome assessment: While objective measurements (MRD1 and PFH) were collected, validated patient-reported outcome measures specific to periocular function and aesthetic satisfaction were not systematically administered. The full impact of the procedure on patient quality of life, functional visual impairment, and psychological distress therefore cannot be fully assessed from these data.

Limited safety data: The absence of adverse events in two cases does not establish the safety profile of this technique. Rare but serious complications (e.g., retrobulbar hemorrhage, globe penetration, orbital compartment syndrome, infection, or permanent visual disturbance) cannot be ruled out without larger prospective cohorts. The predefined stop-rules and safety endpoints represent theoretical safeguards that have not been validated in real-world practice.

Absence of long-term follow-up: While follow-up data at 3–6 months are reported, the durability of effects beyond this period remains unknown. Whether repeated treatments are required, and if so at what interval, has not been investigated.

These limitations collectively indicate that the findings presented herein should be interpreted as preliminary and exploratory. They do not constitute evidence of efficacy or safety sufficient to support routine clinical adoption outside of controlled research settings.

### 5.4 Future directions

These limitations directly chart a course for necessary future research. The imperative next step is the conduct of prospective, controlled, dose-finding trials. Such studies are essential to formally establish the safety profile, determine the minimal effective volume, optimize the treatment schedule (number and interval of sessions), and evaluate comparative effectiveness against standard care or sham procedures. Future research priorities include the following: (1) controlled trials comparing this technique against observation, sham procedure, and standard medical therapy; (2) dose-finding studies to establish the minimal effective volume and optimal treatment schedule; (3) multicenter studies to assess reproducibility across operators and settings; (4) systematic collection of validated patient-reported outcome measures; and (5) larger cohorts to establish a robust safety profile. Furthermore, basic science investigations are warranted to elucidate the biological interactions between dextrose, peripheral nerves, and the neuromuscular junction in the context of neurotoxin exposure. The potential application of this ultrasound-guided hydrodissection approach for managing other BoNT-A-related complications, such as brow ptosis or diplopia, also merits exploration.

## 6 Conclusion

This Methods article presents a novel, standardized protocol for ultrasound-guided preseptal hydrodissection with 5% dextrose for the management of persistent BoNT-A-associated blepharoptosis. The protocol provides a detailed, safety-focused technical guide emphasizing real-time sonographic plane control, vascular mapping with color Doppler, and strict confinement of injectate anterior to the orbital septum.

The application of this protocol in two illustrative cases demonstrated its feasibility and was associated with objective, durable improvement in eyelid position. However, these observations must be interpreted with caution. This work is descriptive and hypothesis-generating, based on a very small sample size (*n* = 2) without a control group. The observed improvements cannot be distinguished from the natural history of spontaneous recovery, and the findings do not establish efficacy, generalizability, or safety.

It is imperative to underscore that this technique is not a first-line intervention. It is positioned strictly as a potential adjunctive option, to be considered only for select patients who remain significantly symptomatic despite exhaustive conventional management, including observation, supportive measures, and trial of topical α-adrenergic agonists. Even within this limited context, the procedure should be undertaken only by practitioners with specialized training in high-frequency orbital ultrasonography and interventional techniques, and ideally within the framework of prospective data collection or clinical trials.

The findings from this protocol description and its initial application are descriptive and hypothesis-generating. They do not establish efficacy or general safety. These observations highlight the necessity for prospective, controlled, dose-finding trials to rigorously determine the procedure’s safety profile, optimal therapeutic parameters, and comparative effectiveness within the established spectrum of care for this complication in aesthetic medicine. Until such evidence is available, this technique should remain investigational.

## Data Availability

The original contributions presented in the study are included in the article/supplementary material. Further inquiries can be directed to the corresponding authors.
